# Mucosal Immunity in COVID-19: A Neglected but Critical Aspect of SARS-CoV-2 Infection

**DOI:** 10.3389/fimmu.2020.611337

**Published:** 2020-11-30

**Authors:** Michael W. Russell, Zina Moldoveanu, Pearay L. Ogra, Jiri Mestecky

**Affiliations:** ^1^ Department of Microbiology and Immunology, Jacobs School of Medicine and Biomedical Sciences, University at Buffalo, Buffalo, NY, United States; ^2^ Department of Microbiology, University of Alabama at Birmingham, Birmingham, AL, United States; ^3^ Division of Infectious Diseases, Department of Pediatrics, Jacobs School of Medicine and Biomedical Sciences, University at Buffalo, Buffalo, NY, United States

**Keywords:** COVID-19, SARS-CoV-2, mucosal immunity, immune response, immunoglobulin A

## Abstract

The mucosal immune system is the largest component of the entire immune system, having evolved to provide protection at the main sites of infectious threat: the mucosae. As SARS-CoV-2 initially infects the upper respiratory tract, its first interactions with the immune system must occur predominantly at the respiratory mucosal surfaces, during both inductive and effector phases of the response. However, almost all studies of the immune response in COVID-19 have focused exclusively on serum antibodies and systemic cell-mediated immunity including innate responses. This article proposes that there is a significant role for mucosal immunity and for secretory as well as circulating IgA antibodies in COVID-19, and that it is important to elucidate this in order to comprehend especially the asymptomatic and mild states of the infection, which appear to account for the majority of cases. Moreover, it is possible that mucosal immunity can be exploited for beneficial diagnostic, therapeutic, or prophylactic purposes.

## Introduction

Although the COVID-19 pandemic has been ongoing now for several months, very little attention has been given to mucosal immunity in SARS-CoV-2 infection. Yet this virus primarily infects the mucosal surfaces of the respiratory tract (and possibly also the digestive tract) at least until advanced stages of the disease when viral RNA may become detectable in the circulation (1). The virus may also be acquired through the mouth, and at the conjunctival surface of the eye whence it drains into the nasal passages through the lacrimal duct. This means that its interactions with the immune system, during both inductive and effector phases, must first occur predominantly if not exclusively at the respiratory and oral mucosae. This has profound implications for the outcomes and should guide our approach to investigating and comprehending adaptive immunity in COVID-19 disease, including its diagnosis, treatment, and effective vaccine development. In terms of both the deployment of immune cells and the production of immunoglobulins, the mucosal immune system is by far the largest component of the entire immune system, having evolved to provide protection at the main sites of infectious threat: the mucosae (2). Secretory IgA (SIgA) is produced in quantities far exceeding those of all other immunoglobulin isotypes combined (3). Together with the serum counterpart, which is derived from a distinct source, the bone marrow, IgA is the most heterogeneous of immunoglobulin isotypes, occurring in three molecular forms (secretory, polymeric, and monomeric), two subclasses (IgA1 and IgA2), and numerous glycoforms (4), collectively indicating marked differences in physiological function relating partly to the locations in which they occur. Whereas circulating IgA is mostly monomeric, and consists predominantly of IgA1 subclass, SIgA is dimeric and consists of variable proportions of IgA1 and IgA2 (**Table 1**). Few functional differences have been attributed to the IgA subclasses, apart from their being preferentially induced by protein vs. carbohydrate antigens (**Table 1**), and the longer hinge of IgA1 gives it greater flexibility to reach separated antigenic epitopes. However, different effector functions of IgA subclasses have been ascribed to their different glycosylation profiles (5).

**Table 1:** Independence of systemic and mucosal IgA compartments.

	Serum	Secretions
Levels	0.5–3.5 mg/ml	Highly variable in different secretions
Maturation	Adult levels reached in adolescence	Adult levels reached at 6–12 months
	**Independent maturation**	
Site of production	Bone marrow >> spleen, lymph nodes	Mucosal tissues
	**IgA produced systemically is not transported into secretions**	
Molecular forms	Predominantly monomeric	Polymeric (dimers and tetramers) SIgA
Subclasses	85% IgA1 15% IgA2	IgA1 dominant in most secretions except for the large intestine and genital tract
Specificity of IgA antibodies		
Proteins	IgA1	IgA1
Polysaccharides	IgA2>IgA1	IgA2>IgA1
Viruses	IgA1	IgA1
Effector functions		Inhibition of antigen uptake
	Anti-inflammatory activity	Inhibition of bacterial adherence
	Neutralization of biologically active antigens	Neutralization of biologically active antigens
		Intracellular neutralization of viruses within epithelial cells
	**Structural and functional differences**	

## The Role of Mucosal Immune Responses

As SARS-CoV-2 first mainly infects the upper respiratory tract (URT), mucosal immune responses are expected to be induced in the nasopharynx, both across the nasal epithelium and *via* the tonsils and adenoids, which are collectively referred to as nasopharynx-associated lymphoid tissue (NALT) that serve as inductive sites for the mucosal immune system (6, 7). It is possible that responses might also be induced through mucosal inductive sites in the lacrimal duct (8) or the oral cavity (9), although the quantitative contribution of such sites to mucosal immune responses in humans is uncertain. Bronchus-associated lymphoid tissue (BALT) is not normally present in adult humans, but can be found in children and adolescents, and may be induced to form by infections (10). This raises interesting questions as to whether responses induced in BALT might contribute to the reported greater resistance of young people to COVID-19 disease, or whether BALT might be induced by SARS-CoV-2 with consequences for the course of infection. All such mucosal inductive site tissues generate IgA-producing mucosal B cells that home to various remote mucosal effector sites where they differentiate into polymeric (p) IgA-secreting plasma cells. In addition, systemic IgG-producing B cells are also induced in the tonsils and these home to peripheral lymphoid tissues where they differentiate and secrete IgG for the circulation (11). In the subepithelial spaces of the mucosae and associated glands, mucosal plasma cells produce pIgA which is selectively transported into the secretions by the polymeric immunoglobulin receptor-mediated pathway, being released as SIgA (12). Both in the nasal passages and as it descends into the trachea and bronchi, the virus encounters a SIgA-dominated environment, which is generated through the mucosal immune system and maintains an essentially non-inflammatory milieu. However, once it reaches the terminal airways and alveoli it enters an environment dominated by IgG derived from the circulation.

Most attention has been given to virus-neutralizing antibodies, especially circulating antibodies (13–15). However, these can only be effective in the prevention of infection or disease if they reach the mucosal surfaces where the virus is present, and it should be noted that circulating IgA, even in polymeric form, is not effectively transported into secretions (16). While plasma-derived IgG occurs in the URT and especially the lower respiratory tract (LRT), IgG is inflammatory in its mode of action, by the induction of such effector mechanisms as complement activation and the engagement of phagocytes such as macrophages and neutrophils as well as natural killer (NK) cells. The serious pathology of COVID-19 occurs in the terminal airways of the lungs, where circulating IgG is the dominant immunoglobulin. The resulting intense inflammation involves multiple molecular and cellular factors, including cells recruited by virus-induced chemo-attractants (17). The cellular arm of the adaptive immune response, including CD4+ and cytotoxic CD8+ T cells, is also delivered *via* the circulation and can reach the alveoli. However, cytotoxic cells by their nature cannot *prevent* infection: they destroy *already infected* cells and thereby curtail further propagation of the infection.

Almost all efforts at vaccine development against COVID-19 focus on systemic injection, which predominantly induces circulatory IgG antibodies and, potentially, cytotoxic T cells (18). These routes are poorly effective at generating mucosal immune responses, which can only be induced by mucosal routes of immunization, including through the NALT in the URT. Mucosal immune responses are partly compartmentalized, as the distribution of the responses depends on the actual route of induction (7, 19). For example, the enteric route predominantly generates responses in the gastro-intestinal tract, whereas the nasal route predominantly generates responses in the respiratory tract and salivary glands (7). The reasons for these differential distributions lie in the imprinting of the T and B cells induced in the respective inductive sites, the gut-associated lymphoid tissues (GALT, such as the intestinal Peyer’s patches) or NALT, with “homing” receptors including specific integrins and chemokine receptors specific for the target tissues (20). In practical terms this means that intranasal immunization should be an effective means of generating predominantly SIgA antibody responses in the URT and LRT, where SARS-CoV-2 could be neutralized and eliminated without inflammatory consequences. In addition, it implies that assaying IgA antibodies in nasal secretions or saliva should be a more informative way of assessing effective immune responses against SARS-CoV-2, whether induced by the natural infection or by intranasal immunization. Assaying serum IgA antibodies, while of additional interest, is *not* a substitute, because serum IgA comes from a different source (mainly the bone marrow) and consists mostly of monomeric IgA1. This is distinct from mucosal SIgA, which consists of both subclasses and is locally synthesized by pIgA-secreting plasma cells resident in the sub-epithelial spaces (lamina propria) of mucosal tissues and glands (21).

SIgA antibodies are known to be effective against various pathogens, including viruses, by such mechanisms as neutralization, inhibition of adherence to and invasion of epithelial cells, agglutination and facilitation of removal in the mucus stream (22). Intracellular mechanisms of inhibiting viral replication have also been described (23). Moreover, SIgA is essentially non-inflammatory, even anti-inflammatory, in its mode of action. IgA does not activate complement by the classical pathway and alternative pathway activation by IgA is largely artifactual, while the lectin pathway depends on the terminal sugar residues in the glycan structures (22). Furthermore, IgA antibodies have even been shown to inhibit complement activation mediated by IgM or IgG antibodies (24, 25). Interestingly, results from a human systemic HIV immunization trial suggest that high levels of serum IgA responses compromised the protective function of IgG antibodies with the same antigenic specificity and were associated with a higher risk of HIV infection (26).

It is noteworthy that whereas mucosal SIgA levels rise rapidly in infants and reach adult levels early in childhood, serum IgA levels mature much more slowly and may not attain full adult levels until adolescence (27). Given the apparent difference in susceptibility to COVID-19 disease between young children and adults (28), these differences in immune response maturation should be considered.

Selective IgA deficiency is relatively common among people of European descent, where prevalence may reach 1 in approximately 400 persons (29). The deficiency affects both mucosal and circulatory compartments and subjects often show increased susceptibility to URT infections. Whether and how IgA-deficiency affects COVID-19 is completely unknown. On one hand, if mucosal SIgA antibodies in the URT exert a protective effect against the early stages of SARS-CoV-2 infection, then deficiency of SIgA would be expected to enhance the infection, facilitating descent into the LRT and leading to advanced disease. Alternatively, an absence of circulatory IgA with its capacity to interfere with IgM- and IgG-mediated defense mechanisms might prove beneficial in LRT infection, where IgG antibodies predominate. Yet again, the absence of anti-inflammatory control by IgA antibodies might facilitate dysregulated inflammation. Thus IgA deficiency affords an opportunity for testing hypotheses concerning the role of IgA in COVID-19.

## Discussion

We contend that mucosal immunity has a major part to play in COVID-19, at several levels. Given that SARS-CoV-2 first infects mainly through the nasal passages, possibly through the eyes followed by drainage into the URT, also through the mouth, we would predict that the first immune responses should be revealed through the mucosal immune system, with the appearance of SIgA antibodies in URT secretions, and also in saliva and lacrimal fluid. Concomitant production of serum IgG and IgA antibodies might also occur (11). Prior to the production of SIgA antibodies, there should be a wave of IgA antibody-secreting cells in the circulation. This typically occurs with a peak at around 6-10 days after a discrete mucosal immunization event, as the B cells induced in the mucosal immune inductive sites (such as NALT) express mucosal homing receptors such as α4β7 integrin and migrate to the effector sites where they terminally differentiate into pIgA-secreting plasma cells (11, 30). Circulating IgG antibody-secreting cells are also induced by antigens stimulating responses in the tonsils, and these usually express peripheral homing receptors, such as L-selectin. The period in which these cells can be detected is limited, as this wave of migrating B cells is transient after an inducing event, which in the case of SARS-CoV-2 infection might be up to 4-5 days (or longer) before symptoms first occur. If so, peak cell migration could be some 4-5 days after symptoms occur. However, in the presence of continuing infection and ongoing immune stimulation, it is possible that antibody-secreting cells would continue to circulate as repeated waves of induced B cells are released from the inductive sites, as has been reported for HIV infections (31). Because of uncertainties in the kinetics of IgM, IgG, and IgA antibody development in infected individuals, detailed analysis of antibody-secreting cells and their expression of homing receptors, and their persistence in lymphoid tissues, will provide essential additional and more comprehensive information concerning the immune response to SARS-CoV-2. Such analyses might illuminate differences in the clinical outcome of the infection in children compared to symptomatic or asymptomatic adults. Determining these antibody and cellular responses of the mucosal immune system can be expected to provide valuable information that is distinct from, and complementary to, the determination of serum IgG antibody responses. Furthermore, it is reasonable to expect that the information would yield valuable insights into the progress of COVID-19 disease, whether it is confined to the URT and resolved with minimal symptoms, as seems to be the case in the majority of infections that are asymptomatic or clinically mild, or descends into the LRT with more severe consequences leading to advanced, and possibly fatal, inflammatory disease in the terminal airways (32). However, a key requirement for obtaining reliable results is that procedures used for the collection of samples as well as the assay methods must be designed with the characteristics of mucosal immune responses and the distinctive features of secretions compared to serum in mind (33).

It is noteworthy that IgA antibody responses have been recorded in nasal fluids of volunteers infected with the common cold coronavirus 229E, and were associated with shortened periods of viral shedding (34). Serum and salivary IgA antibody responses to SARS-Cov-2 spike antigens have now been reported (35), and salivary IgA antibodies persisted for at least 3 months. Interestingly, while good correlations were found between serum and salivary IgM and IgG antibody levels, there was much weaker correlation between serum and salivary IgA antibodies, as expected given that salivary IgM and IgG are largely derived from the circulation, whereas salivary IgA is mostly generated locally in the salivary glands as SIgA (19). Two recent preprints report that IgA antibodies against SARS-CoV-2 were elevated in nasal fluids, tears, and saliva of infected subjects (36, 37), and that IgA-secreting plasmablasts expressing the mucosal chemokine receptor CCR10 were elevated in the peripheral blood of SARS-CoV-2-infected subjects (37). These findings support the concept that mucosal IgA antibody responses are induced by SARS-CoV-2. Further studies aimed at relating these responses to the course of infection in subjects of different age-groups and with different disease outcomes are awaited with great interest.

Finally we expect that efforts in vaccine development aimed at inducing mucosal immune responses and memory cells, especially in the URT, would yield benefits not seen with conventional parenteral routes of vaccine administration. Intranasal vaccines are already available against influenza and others are under development (30, 38). The advantages, in addition to needle-free administration, include the generation of both mucosal (SIgA) and circulating (IgG and IgA) antibodies, as well as T-cell responses. As discussed above, such responses might achieve desirable results not obtained with systemic immunization routes.

In summary, based on the route whereby SARS-CoV-2 infection is acquired and the independence of mucosal and systemic responses, there must be a mucosal immune dimension to COVID-19. Whether it makes a significant contribution to the outcome of SARS-CoV-2 infection, or can be exploited to good effect for diagnostic purposes or for therapy and prophylaxis, can only be determined by carrying out appropriate investigations. **Table 2** lists some potentially important studies that should be undertaken to elucidate mucosal immune responses in SARS-CoV-2 infection.

**Table 2:** Desirable future studies on mucosal immune responses to SARS-CoV-2.

• Determine mucosal and serum IgA, IgG, and IgM antibody responses to SARS-CoV-2 through all states of infection and disease, and in response to vaccines • Determine the molecular form and subclass of IgA antibodies in relation to COVID-19 disease stage and the effector functions of IgA • Compare mucosal and serum IgA antibody responses to SARS-CoV-2 across different age groups • Determine the characteristics of IgA antibody-secreting and memory B cells induced by SARS-CoV-2 infection or vaccination and their homing potential for mucosal or systemic tissues • Determine the characteristics of T cells induced by SARS-CoV-2 (or by vaccines) and their homing potential for mucosal or systemic tissues

## Data Availability Statement

Any original contributions presented in the study are included in the article material. 

## Author Contributions

The aims of this article were conceived by all authors. MR wrote the first draft, which was reviewed and amended by all authors to create the final version. All authors contributed to the article and approved the submitted version.

## Conflict of Interest

The authors declare that the research was conducted in the absence of any commercial or financial relationships that could be construed as a potential conflict of interest.
